# Obstructive Sleep Apnea Following Bariatric Surgery: 20 Year Outcomes From the Swedish Obese Subjects Study

**DOI:** 10.1002/oby.70154

**Published:** 2026-02-25

**Authors:** Ida Arnetorp, Markku Peltonen, Kajsa Sjöholm, Per‐Arne Svensson, Peter Jacobson, Magdalena Taube, Lena M. S. Carlsson, Johanna C. Andersson‐Assarsson, Sofie Ahlin

**Affiliations:** ^1^ Department of Molecular and Clinical Medicine Institute of Medicine, Sahlgrenska Academy, University of Gothenburg Gothenburg Sweden; ^2^ Finnish Institute for Health and Welfare Helsinki Finland; ^3^ Institute of Health and Care Sciences, Sahlgrenska Academy, University of Gothenburg Gothenburg Sweden; ^4^ Department of Clinical Physiology Region of Västra Götaland, NU Hospital Group Trollhättan Sweden

**Keywords:** bariatric surgery, obesity, obstructive sleep apnea (OSA)

## Abstract

**Objective:**

Bariatric surgery has been suggested to improve obstructive sleep apnea (OSA) in short‐term studies, but long‐term evidence is limited. We evaluated remission and new onset of OSA over 20 years in participants from the Swedish Obese Subjects (SOS) study, comparing bariatric surgery with usual obesity care.

**Methods:**

The SOS study is a nonrandomized, controlled intervention study including 4047 individuals who received bariatric surgery (*n* = 2010) or usual obesity care (*n* = 2037). OSA status was assessed via questionnaires at baseline and at 1, 2, 3, 4, 6, 8, 10, 15, and 20 years. We examined remission and new onset of OSA among participants with or without the condition at baseline, comparing surgery to usual care.

**Results:**

On average over 20 years, bariatric surgery was associated with a 32.1 percentage points lower prevalence of OSA in participants with baseline OSA (95% CI: −36.9 to −27.2; *p* < 0.001) compared to usual care. Among those without baseline OSA, surgery patients had a 5.8 percentage points lower prevalence of new‐onset OSA (95% CI: −7.1 to −4.6; *p* < 0.001).

**Conclusions:**

Bariatric surgery was associated with durable OSA benefits, including higher remission and lower long‐term new onset of OSA compared to usual obesity care.

**Trial Registration:**

ClinicalTrials.gov identifier: NCT01479452

## Study Importance

- **What is already known?**
  ○ Several short‐ and mid‐term studies, including our own previous findings, indicate a strong association between bariatric surgery and resolution of obstructive sleep apnea (OSA) or improvement of apnea/hypopnea index, but long‐term studies are lacking.
- **What does this study add?**
  ○ This study extends existing knowledge by suggesting that the association between bariatric surgery and remission of OSA is maintained over an exceptionally long follow‐up period of 20 years, compared to a matched control group.
  ○ Moreover, it indicates that bariatric surgery is associated with a reduced long‐term incidence of de novo OSA among individuals without baseline OSA, compared to usual obesity care.
- **How might these results change the direction of research or the focus of clinical practice?**
  ○ These findings point toward sustained benefits of bariatric surgery in both the resolution of existing OSA and the prevention of new cases.
  ○ This provides support for broader consideration of bariatric surgery as both a therapeutic and preventive strategy for OSA in individuals with obesity.

## 1 Introduction

Obesity is a well‐established risk factor for obstructive sleep apnea (OSA) [1] and the prevalence of OSA has increased in parallel with rising obesity rates [2]. The pathophysiology of OSA is considered multifactorial and one suggested mechanism is increased fat deposition in the upper airway [3]. Neck circumference can be used as a predictor for OSA [4] and is included as a variable in screening instruments for OSA [5].

OSA is characterized by recurrent episodes of upper airway collapse and obstruction during sleep, frequently resulting in brief awakenings (arousals) or oxygen desaturation events, which leads to fragmented sleep [6]. Classic symptoms of OSA, such as loud snoring, daytime sleepiness, and breathing pauses, are crucial for diagnosis. However, patient awareness of these symptoms often depends on information from bed partners, and the subjective nature of daytime sleepiness presents challenges for accurate assessment [1].

OSA impairs all aspects of health‐related quality of life [7] and is strongly associated with a range of cardiovascular conditions, including hypertension, heart failure, coronary artery disease, pulmonary hypertension, atrial fibrillation, and stroke [8, 9]. Furthermore, previous studies, including our own, have reported associations between OSA and increased all‐cause mortality [10, 11], as well as an increased risk of traffic accidents [12].

Bariatric surgery promotes substantial and sustainable weight loss while reducing OSA severity and alleviating daytime sleepiness [13]. We have previously reported significant improvements in OSA symptoms 2 years after bariatric surgery in the Swedish Obese Subjects (SOS) study [14], and several other short‐ or mid‐term studies support a strong association between bariatric surgery and resolution or improvement of OSA [15, 16, 17, 18]. However, long‐term evidence is lacking. Therefore, this study aims to assess OSA over a 20‐year period following bariatric surgery or usual obesity care.

## 2 Methods

### 2.1 The SOS Study

The SOS study is a prospective, nonrandomized, controlled intervention study that enrolled 4047 individuals with obesity between 1987 and 2001. Participants either underwent bariatric surgery (surgery group, *n* = 2010) or received usual obesity care (control group, *n* = 2037). The study design has been described in detail previously [19, 20], and the recruitment process is illustrated in Figure S1. Briefly, participants were recruited via mass media campaigns and at primary health care centers. Inclusion criteria were age 37–60 years and a body mass index (BMI) of 34 kg/m^2^ or more for men and 38 kg/m^2^ or more for women. Exclusion criteria were minimal and intended primarily to ensure surgical operability. Notably, the same inclusion and exclusion criteria applied to both groups and all participants were eligible for surgery.

The SOS study's primary endpoint was mortality [19, 21], and OSA was not a predefined secondary endpoint in the original study protocol. Ethical approval was obtained from seven regional ethics review boards in Sweden, and all participants provided oral or written informed consent (ethical approval reference numbers 184‐90 and T508‐17). The study was conducted according to the Declaration of Helsinki. The study is registered at ClinicalTrials.gov identifier NCT01479452.

A total of 25 surgical departments and 480 primary health care centers contributed to the study. Participants in the surgery group elected to undergo surgery and underwent one of three procedures: gastric banding (18%), vertical banded gastroplasty (69%), or gastric bypass (13%). Control group participants received usual obesity care at their primary health care centers. The surgery and control groups were matched using the sequential treatment assignment method [22] based on 18 variables, including demographic data, anthropometric measurements, metabolic indicators, comorbid conditions, and psychosocial factors [20]. The entire matching process was computerized, ensuring no investigator involvement.

The intervention was considered to begin on the day of surgery for both the surgery participants and their matched controls. All participants underwent a baseline examination 4 weeks prior to the intervention start. Follow‐up visits for both surgery and control participants, including physical examinations and questionnaires, were conducted at 0.5, 1, 2, 3, 4, 6, 8, 10, 15, and 20 years after study inclusion.

### 2.2 Obstructive Sleep Apnea and Sleep Apnea Risk Factors

Information on sleep apnea and associated risk factors was collected through study questionnaires and physical examinations. Key risk factors assessed included age, sex, and anthropometric measurements. In the current report, individuals with occurrence of frequent, observed apneas, assessed through the question “Have relatives or others noticed that you often take short breathing pauses during sleep?” were considered to have OSA. Participants provided a “yes” or “no” response.

### 2.3 Follow‐Up

This report includes data collected at baseline and during follow‐up visits at 1, 2, 3, 4, 6, 8, 10, 15, and 20 years after study entry.

### 2.4 Statistical Analysis

Baseline comparisons between the surgery and control groups were performed using Student's *t*‐test for continuous variables and Fisher's exact test for categorical variables. Results are presented as means with standard deviations (SD) or as counts and percentages for categorical variables.

Changes in BMI over time were analyzed with multilevel mixed‐effects regression models.

For the analysis of OSA incidence and remission, both the surgery and control groups were subdivided based on the presence or absence of OSA at baseline. The proportions of participants reporting OSA during follow‐up at 1, 2, 3, 4, 6, 8, 10, 15, and 20 years were calculated within each subgroup. Differences in these proportions over time were analyzed using random‐effects logistic regression models for binary outcomes. Observations were considered nested within individuals, and all statistical tests, including confidence intervals (CI), accounted for the repeated‐measures structure of the data. Differences in proportions between the groups are expressed as percentage points over the entire follow‐up period.

Association between OSA status and changes in anthropometric measurements at the 2‐ and 10‐year follow‐ups were analyzed with linear regression models. Analysis of changes in BMI over time was adjusted for sex and baseline age. All other analyses were adjusted for sex, baseline age, BMI, smoking, and year of inclusion.

Statistical analyses were conducted on a per‐protocol basis. Three participants who initially opted for bariatric surgery did not undergo the surgical procedure and were included in the control group in the current analysis (*n* = 2040). In addition, all participants were included in their original study group until bariatric surgery was performed in the control group (303 out of 2040, 14.9%) or an operation which reinstated normal anatomy was performed in the surgery group (102 out of 2007, 5.1%), at which point they were censored from the analyses. For individuals who dropped out of the study, we assumed missing data were “missing at random” (i.e., missing data are only dependent on the participant's observed data), and all the observed data were used in the analyses. To assess the potential impact of missing data, sensitivity analyses using multiple imputation were conducted. The imputation model included all available sleep apnea data from the follow‐up period, with baseline variables (age, sex, BMI, smoking status, and treatment group [surgery or control]) used as predictors for missing values. A total of 30 imputed datasets were generated. Sensitivity analyses were also conducted based on participants' relationship status at baseline.

All statistical tests were two‐tailed and *p* values of less than 0.05 were considered statistically significant. Stata software, version 18.0 (StataCorp LLC, College Station, TX) was used for analyses.

## 3 Results

### 3.1 Baseline Characteristics of the Study Cohort

Baseline characteristics of participants with and without OSA in the surgery group (without OSA: *n* = 1505; with OSA: *n* = 500) and control group (without OSA: *n* = 1582; with OSA: *n* = 453) are presented in Table 1. Overall, participants in the surgery group were younger and had higher BMI, as well as larger neck and waist circumferences compared to those in the control group—regardless of baseline OSA status. Additionally, the surgery group had higher levels of total cholesterol and a higher prevalence of hypertension and type 2 diabetes compared to controls, regardless of baseline OSA status.

**TABLE 1:** Baseline characteristics of the SOS study cohort in participants with and without OSA at baseline.

	Without OSA at baseline		OSA at baseline	
	Control	Surgery	Control	Surgery
*n*	1582	1505	453	500
Age (years)	48.6 (6.3)	47.3 (6.0)	49.2 (6.0)	47.1 (5.8)
Male sex, *n* (%)	362 (23)	330 (22)	230 (51)	257 (51)
Married/partner, *n* (%)	1188 (75)	1092 (73)	346 (76)	372 (74)
BMI^a^	40.1 (4.8)	42.4 (4.4)	40.2 (4.5)	42.3 (4.8)
Neck circumference (cm)	42.3 (4.0)	43.1 (4.1)	45.1 (4.6)	45.8 (4.7)
Waist circumference (cm)	119.4 (11.3)	125.1 (10.8)	123.3 (10.7)	127.9 (11.3)
Fasting blood glucose (mmol/L)	4.9 (1.8)	5.1 (1.9)	5.1 (1.9)	5.4 (2.2)
Insulin (mU/L)	17.3 (10.5)	20.6 (12.8)	20.6 (13.8)	24.3 (16.0)
HOMA‐IR	4.5 (3.9)	5.5 (5.2)	5.5 (5.6)	6.8 (5.9)
Type 2 diabetes^b^, *n* (%)	224 (14)	268 (18)	81 (18)	124 (25)
Total cholesterol (mmol/L)	5.6 (1.1)	5.8 (1.1)	5.7 (1.0)	5.9 (1.1)
HDL cholesterol (mmol/L)	1.4 (0.3)	1.4 (0.3)	1.3 (0.3)	1.3 (0.3)
LDL cholesterol (mmol/L)	3.3 (0.9)	3.5 (1.0)	3.5 (0.9)	3.5 (1.0)
Daily smoking, *n* (%)	300 (19)	375 (25)	120 (27)	143 (29)
Cardiovascular disease before baseline, *n* (%)	27 (2)	29 (2)	22 (5)	17 (3)
Hypertension^c^, *n* (%)	976 (62)	1166 (78)	321 (71)	403 (81)

*Note*: Data are presented as mean (SD) or as number (%).

^a^ Body mass index: weight in kilograms divided by height in meters squared.

^b^ Type 2 diabetes: HbA1c ≥ 48 mmol/mol or fasting blood glucose ≥ 6.1 mmol/L (≥ 110 mg/dL) or self‐reported diabetes medication use.

^c^ Hypertension: diastolic blood pressure > 90 mmHg, systolic blood pressure > 140 mmHg, or self‐reported antihypertensive medication use.

### 3.2 Follow‐Up

At the time of their 20‐year follow‐up examination, 1374/2040 (67%) persons in the control group and 1429/2007 (71%) in the surgery group were still alive. Of those, 521 (38%) and 621 (43%) persons in the control and surgery groups, respectively, participated in the 20‐year follow‐up. The number of participants with available OSA data at each time point, after censoring for treatment change and stratified by baseline OSA status, is presented in Table S1.

### 3.3 Changes in BMI During Follow‐Up

**FIGURE 1:**
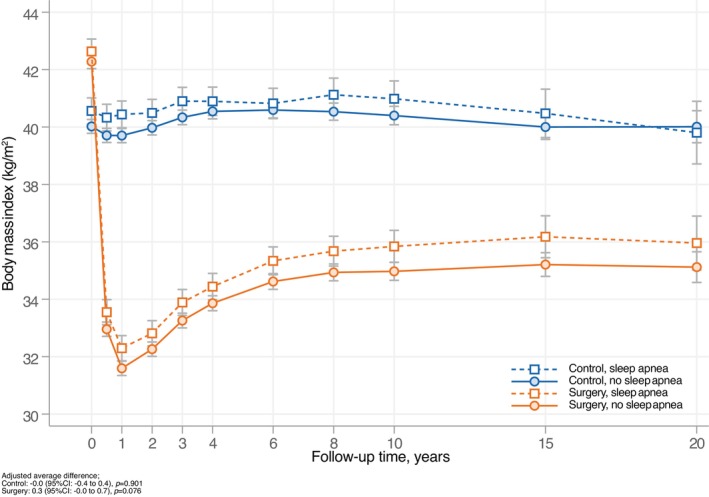
BMI changes over 20 years for surgery and control groups, stratified by OSA at baseline. SD with 95% CI shown as error bars. Adjusted for age at baseline and sex. [Color figure can be viewed at wileyonlinelibrary.com]

Longitudinal changes in BMI for the surgery and control groups, stratified by baseline OSA status, are shown in Figure 1. Over a 20‐year period, bariatric surgery resulted in a substantial and sustained weight reduction when compared to usual care. However, BMI trajectories did not differ significantly between participants with and without baseline OSA, in either the surgery group (*p* = 0.076) or the control group (*p* = 0.901).

### 3.4 Remission of OSA After Bariatric Surgery

**FIGURE 2:**
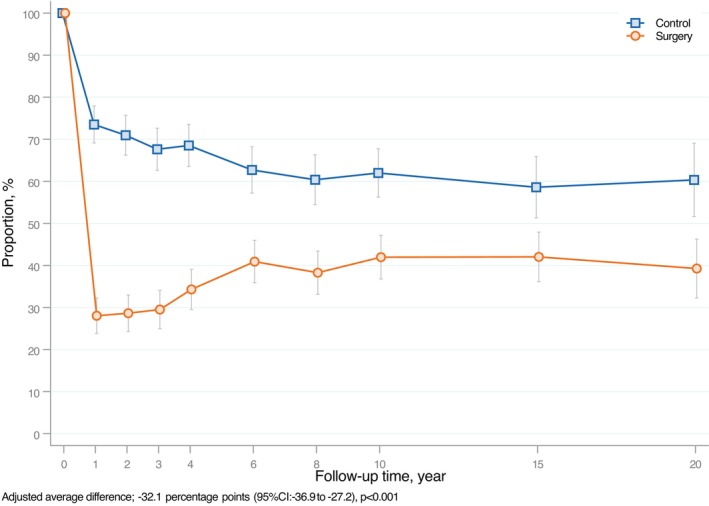
Remission of OSA in individuals with OSA at baseline in the surgery and control groups of the SOS study. Adjusted proportions and 95% CI for OSA at each follow‐up time point, derived from random‐effect logistic regression model analyses adjusted for baseline age, sex, BMI, daily smoking, and inclusion year. [Color figure can be viewed at wileyonlinelibrary.com]

Among participants with OSA at baseline, those who underwent bariatric surgery showed significantly lower prevalence of OSA over 20 years compared to those receiving usual obesity care (adjusted average difference −32.1 percentage points; 95% CI: −36.9 to −27.2; *p* < 0.001; Figure 2). Results from sensitivity analyses with multiple imputation of missing data showed similar results as the main analyses (adjusted average difference −29.1 percentage points; 95% CI: −33.9 to −24.2; *p* < 0.001). At the 2‐year follow‐up, 71.4% of individuals in the surgery group were in remission from OSA, compared to 29.0% in the control group. Following this initial remission, a small increase in the proportion of individuals reporting OSA was observed in the surgery group, but levels stabilized after 6 years. By the end of follow‐up, 60.7% of the individuals in the surgery group were in remission of OSA, compared to 39.7% in the control group. Subgroup analyses by sex showed consistent results: both men and women in the surgery group had lower prevalence of OSA over 20 years than their counterparts in the control group (adjusted average difference −34.6 percentage points; 95% CI: −41.2 to −28.1, and −29.6 percentage points; 95% CI: −36.8 to −22.4, for men and women, respectively, *p* < 0.001 for both; Figures S2A and S2B). Sensitivity analyses based on participants' relationship status at baseline displayed similar results as the main analysis (data not shown).

### 3.5 Changes in Anthropometry and OSA Remission

Among participants in the surgery group with OSA at baseline, those who achieved remission at the 2‐ or 10‐year follow‐ups showed more favorable changes in anthropometric measures—including BMI, body weight, and neck and waist circumferences—compared to those who continued to report OSA at the same time points, in adjusted analyses (Table 2). In contrast, among participants in the control group, anthropometric changes did not differ significantly between those with or without OSA remission at the 2‐ or 10‐year follow‐ups.

**TABLE 2:** Changes in anthropometric measurements in participants with OSA at baseline, stratified by OSA status (remission vs. persistent OSA) at year 2 and year 10, analyzed with linear regression models.

Year 2	Surgery group			Control group		
	OSA remission, *n* = 304	Persistent OSA, *n* = 117	Adjusted *p*	OSA remission, *n* = 99	Persistent OSA, *n* = 247	Adjusted *p*
2‐year change BMI (kg/m^2^)	−10.7 (5.4)	−7.8 (4.0)	< 0.001	−0.1 (2.9)	0.1 (2.7)	0.385
2‐year change weight (kg)	−31.7 (16.0)	−23.4 (11.6)	< 0.001	−0.5 (8.9)	0.4 (7.8)	0.281
2‐year change neck circumference (cm)	−5.3 (3.4)	−4.3 (2.8)	0.033	−0.3 (2.4)	−0.2 (2.2)	0.543
2‐year change waist circumference (cm)	−22.7 (12.4)	−18.9 (10.5)	0.034	−0.4 (7.4)	−0.4 (6.9)	0.827

Year 10	OSA remission, *n* = 186	Persistent OSA, *n* = 130	Adjusted *p*	OSA remission, *n* = 91	Persistent OSA, *n* = 148	Adjusted *p*
10‐year change BMI (kg/m^2^)	−8.1 (5.5)	−4.9 (4.7)	< 0.001	0.5 (4.3)	1.2 (4.1)	0.223
10‐year change weight (kg)	−25.0 (15.8)	−15.2 (13.8)	< 0.001	0.7 (12.9)	3.1 (12.8)	0.147
10‐year change neck circumference (cm)	−4.1 (3.7)	−2.2 (3.7)	< 0.001	0.0 (2.9)	0.4 (3.1)	0.297
10‐year change waist circumference (cm)	−15.7 (12.7)	−10.5 (12.7)	0.003	2.0 (9.6)	3.8 (9.7)	0.086

*Note*: Data are presented as mean (SD). Analyses are adjusted for baseline age, sex, BMI, daily smoking, and inclusion year.

### 3.6 New Onset of OSA After Bariatric Surgery

**FIGURE 3:**
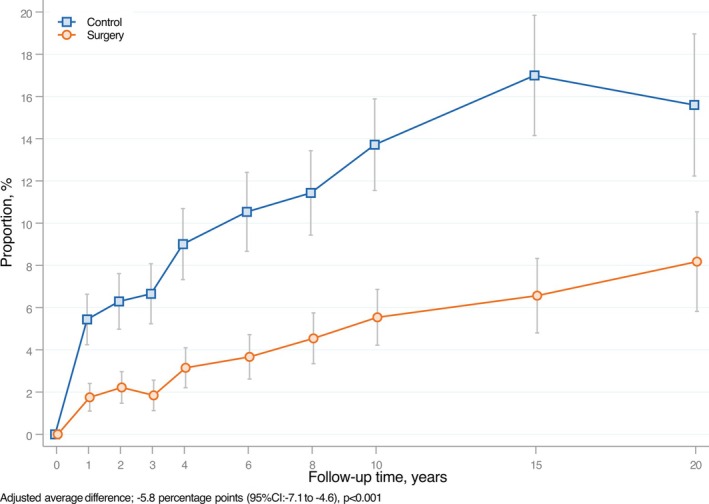
New‐onset OSA in individuals without OSA at baseline in the surgery and control groups of the SOS study. Adjusted proportions and 95% CI for OSA at each follow‐up time point, derived from random‐effect logistic regression model analyses, adjusted for baseline age, sex, BMI, daily smoking, and inclusion year, among participants without OSA at baseline. [Color figure can be viewed at wileyonlinelibrary.com]

Among participants without OSA at baseline, the prevalence of OSA over the 20‐year follow‐up period was significantly lower in the surgery group compared to the control group (adjusted average difference −5.8 percentage points; 95% CI: −7.1 to −4.6; *p* < 0.001). Results from sensitivity analyses with multiple imputation of missing data showed similar results as the main analyses (adjusted average difference −7.0 percentage points; 95% CI: −8.5 to −5.5; *p* < 0.001). Although the prevalence of OSA increased gradually over time in both groups, the control group consistently showed higher proportions of participants with OSA throughout the follow‐up period. The largest difference between groups was observed at the 15‐year follow‐up (Figure 3), when 6.6% of the participants in the surgery group reported OSA, compared to 17.0% in the control group. Sex‐stratified analyses showed similar results, with both men and women in the surgery group displaying a significantly lower prevalence of OSA compared to usual care controls (adjusted average difference −8.2 percentage points [95% CI: −11.4 to −5.0] in men and −5.3 percentage points [95% CI: −6.6 to −4.0] in women, *p* < 0.001 for both; Figures S3A and S3B). Sensitivity analyses based on participants' relationship status at baseline displayed similar results as the main analysis (data not shown).

### 3.7 Changes in Anthropometry and New Onset of OSA

In the surgery group, participants with new‐onset OSA by the 2‐year follow‐up displayed more modest reductions in BMI, weight, and neck and waist circumferences between baseline and year 2 compared to those who remained free of OSA, in adjusted analyses (Table 3). In contrast, among control group participants, only the reduction in waist circumference differed significantly between those with and without incident OSA. At the 10‐year follow‐up, in both the surgery and control groups, participants with new‐onset OSA showed less favorable changes in BMI, body weight, and neck and waist circumferences, compared to those who remained free of OSA (Table 3).

**TABLE 3:** Changes in anthropometric measurements in participants without OSA at baseline, stratified by OSA status (free of OSA vs. new‐onset OSA) at year 2 and year 10, analyzed with linear regression models.

Year 2	Surgery group			Control group		
	Free of OSA, *n* = 1332	New‐onset OSA, *n* = 34	Adjusted *p*	Free of OSA, *n* = 1181	New‐onset OSA, *n* = 83	Adjusted *p*
2‐year change BMI (kg/m^2^)	−10.1 (4.8)	−8.4 (5.4)	0.006	−0.0 (3.0)	0.3 (2.9)	0.121
2‐year change weight (kg)	−28.5 (13.7)	23.9 (14.8)	0.003	−0.1 (8.6)	1.0 (9.0)	0.067
2‐year change neck circumference (cm)	−4.5 (3.1)	−3.7 (2.9)	0.033	−0.2 (2.1)	0.2 (2.5)	0.056
2‐year change waist circumference (cm)	−21.6 (12.4)	−18.1 (13.6)	0.034	0.1 (8.3)	1.8 (8.2)	0.031

Year 10	Free of OSA, *n* = 1010	New‐onset OSA, *n* = 63	Adjusted *p*	Free of OSA, *n* = 805	New‐onset OSA, *n* = 119	Adjusted *p*
10‐year change BMI (kg/m^2^)	−7.4 (5.2)	−4.5 (5.0)	< 0.001	0.8 (4.6)	2.5 (4.2)	< 0.001
10‐year change weight (kg)	−21.5 (14.8)	−13.9 (14.3)	< 0.001	1.5 (13.0)	6.2 (12.8)	< 0.001
10‐year change neck circumference (cm)	−3.4 (3.1)	−2.1 (3.4)	0.001	0.3 (2.7)	1.0 (2.7)	0.021
10‐year change waist circumference (cm)	−14.9 (13.4)	−6.6 (11.8)	< 0.001	2.8 (11.1)	7.1 (10.8)	< 0.001

*Note*: Data are presented as mean (SD). Analyses are adjusted for baseline age, sex, BMI, daily smoking, and inclusion year.

## 4 Discussion

In this study, we examined OSA following bariatric surgery using data from the large, prospective, controlled SOS study, which includes over 4000 participants and provides repeated follow‐up assessments for up to 20 years. Our findings indicate that, compared to usual obesity care, bariatric surgery is associated with a higher long‐term remission rate of OSA in participants with OSA at baseline, as well as a lower prevalence of OSA in those without baseline OSA. Among participants in the surgery group, those who achieved remission of OSA or did not develop new‐onset OSA exhibited greater reductions in anthropometric measures both at 2 and 10 years, compared to those who either did not achieve OSA remission or who developed new‐onset OSA.

Previously, we [14], and others [23, 24, 25, 26, 27, 28, 29, 30, 31, 32, 33, 34], have reported that bariatric surgery is associated with short‐term remission or improvement of OSA. However, many of these studies are limited by small sample sizes and/or the absence of control groups [23, 24, 25, 26, 27, 28, 29, 32, 34]. We extend these findings with our results from the SOS study which provides robust, long‐term data, suggesting that bariatric surgery is associated with sustained remission of OSA over 20 years with 32 percentage points lower prevalence in the surgery group compared to the control group over time. At the 20‐year follow‐up, 61% of participants in the surgery group had achieved remission from OSA. While a recent meta‐analysis [15] reported similar rates of remission or improvement (64%) following bariatric surgery, the included studies were predominantly short term, with only three extending beyond 5 years—one of which was from the SOS study itself. The SOS study thus provides uniquely long‐term data. Notably, 40% of participants in the control group were classified as being in remission at the 20‐year follow‐up. This finding underscores the importance of including a control group, as it enables consideration of factors such as regression to the mean [35]. The relatively high remission rate among controls may also reflect that all SOS participants had voluntarily applied to the study, indicating strong motivation to lose weight and adopt lifestyle changes. Some control participants achieved weight loss, and those without OSA demonstrated more favorable long‐term weight development compared with individuals with OSA. Although these differences were modest, they likely reflect a generally healthier lifestyle within this subgroup.

The potential of bariatric surgery to prevent OSA has been less thoroughly explored than its effects on remission. In participants free from OSA at study start, we found that bariatric surgery was associated with a reduced risk of developing obstructive sleep apnea over a 20‐year follow‐up period, compared to a control group. In a multicenter study involving 187 patients who underwent laparoscopic Roux‐en‐Y gastric bypass, an 8% incidence of OSA was reported after 12 months postoperatively among participants without OSA at baseline (*n* = 55) [31]. This rate is notably higher than the approximately 2% observed in our study at the 1‐year follow‐up. However, this study did not include a control group, limiting the ability to assess the potential preventive effect of bariatric surgery.

The pathophysiology of OSA is considered multifactorial. Obesity is a well‐established risk factor for OSA, and one suggested mechanism is increased fat deposition in the upper airway leading to its collapse [3]. In line with this, neck circumference has been shown to be a better predictor of OSA than only BMI [4]. Other contributing mechanisms to OSA have also been identified, including craniofacial anatomical variations and unstable ventilatory chemoreflex control [3].

Our findings further underscore the central role of obesity in the development and persistence of OSA. Participants who achieved OSA remission following bariatric surgery had significantly greater reductions in neck and waist circumferences, BMI, and weight at both the 2‐ and 10‐year follow‐ups. Moreover, within the surgery group, participants who remained free of OSA 2 and 10 years after surgery had more pronounced decreases in anthropometric measurements compared to those who developed OSA. These findings are consistent with previous research showing that patients who experienced OSA remission had significantly greater weight loss at 1 year following surgery compared to those who did not achieve remission [18]. The pivotal role of weight loss in OSA remission has also been reinforced by recent evidence showing substantial improvements in OSA severity with pharmacologically induced weight loss using tirzepatide [36]. Tirzepatide has recently been approved by the FDA for the treatment of OSA in adults with obesity [37]. In parallel, lifestyle and dietary interventions that promote weight loss have also been shown to significantly reduce OSA severity, underscoring the multifaceted benefits of weight reduction [38]. However, long‐term data on these nonsurgical interventions remain limited, making findings from bariatric surgery studies, such as the SOS study, particularly valuable for addressing the current knowledge gap regarding the long‐term association between sustained weight loss and OSA outcomes.

The SOS study has both strengths and limitations. It includes a large, well‐characterized surgery cohort and a carefully matched control group. Most notably, to the best of our knowledge, it remains the only study to provide up to 20 years of follow‐up data on sleep apnea outcomes following bariatric surgery, underscoring its unique contribution to the field. The SOS study enrolled participants between 1987 and 2001, and the bariatric procedures performed reflect the standard surgical practices of that era. While some of these procedures are no longer commonly used today, many individuals who underwent them remain patients in the current health care system. Therefore, our findings remain clinically relevant and important to report. The bariatric procedures used in the SOS study differed somewhat in their long‐term weight loss outcomes, with gastric bypass producing the greatest BMI reduction. Notably, vertical banded gastroplasty—the most common surgical method used in the SOS study—has been shown to result in weight loss outcomes comparable to those of sleeve gastrectomy [39], one of the most frequently performed bariatric procedures today. A key limitation is the nonrandomized design. However, a randomized controlled trial was not feasible at the time of study initiation in 1980 due to ethical concerns and fear of high postoperative mortality rates. Another limitation is that OSA was not a predefined endpoint. The evaluations of OSA are based on questionnaire‐derived data rather than polysomnography—the current gold standard for OSA diagnosis. However, a previous meta‐analysis has shown that OSA questionnaires serve as effective screening tools for identifying individuals with OSA [40]. In the present analysis, participants were asked, “Have relatives or others noticed that you often take short breathing pauses during sleep?”—a question that depends on observations from a partner or family member. To address this, a sensitivity analysis based on partnership status at baseline was performed and showed results consistent with those of the main analysis.

## 5 Conclusion

In conclusion, our findings suggest that bariatric surgery is associated with significantly higher rates of OSA remission and a reduced risk of developing OSA compared to usual obesity care over a 20‐year follow‐up period. Furthermore, this study suggests that weight loss and reductions in anthropometric measures likely contribute to the beneficial effects of bariatric surgery on OSA outcomes.

## Author Contributions

Johanna C. Andersson‐Assarsson and Sofie Ahlin had full access to all the data in the study and take responsibility for the integrity of the data and the accuracy of the data analysis. Concept and design: Ida Arnetorp, Markku Peltonen, Johanna C. Andersson‐Assarsson, Sofie Ahlin. Analysis and interpretation of data: All authors. Drafting the article: Ida Arnetorp, Johanna C. Andersson‐Assarsson, Sofie Ahlin. Critical revision of the manuscript for important intellectual content: All authors. Final approval of the article: All authors. Statistical expertise: Markku Peltonen. Obtained funding: Kajsa Sjöholm, Per‐Arne Svensson, Magdalena Taube, Lena M.S. Carlsson, Johanna C. Andersson‐Assarsson, Sofie Ahlin. Administrative, technical, or logistic support: All authors.

## Funding

This work was supported by Health & Medical Care Committee of the Region Västra Götaland (VGFOUFBD‐1004644), the Vetenskapsrådet (2021‐01303, 2021‐01496), the Swedish governmental funding of clinical research (ALF) (ALFGBG‐1005957, ALFGBG‐1006165, ALFGBG‐1006182, ALFGBG‐1006404), the Adlerbert Research Foundation, the Wilhelm and Martina Lundgren foundation (2023‐SA‐4298, 2025‐SA‐4942, 2025‐SA‐4961), and the Alcohol Research Council of the Swedish Retailing Monopoly (2023‐0038).

## Conflicts of Interest

Kajsa Sjöholm: Speaker honoraria from AstraZeneca and Encore Medical Education. Lena M. S. Carlsson: Consultancy for AstraZeneca. The other authors declared no conflicts of interest.

## Supporting information


**Data S1:** Supporting Information.

## Data Availability

The data are subject to legal restrictions according to national legislation. Confidentiality regarding personal information in studies is regulated in the Public Access to Information and Secrecy Act (SFS 2009:400), OSL. There is a possibility to apply to get access to public documents that an authority holds. In this case, the University of Gothenburg is the specific authority that holds the documents. A request to get access to public documents can be rejected or granted with reservations. If the authority refuses to disclose the documents the applicant is entitled to get a written decision that can be appealed to the administrative court of appeal.
