# Supplementary material for: Obstructive Sleep Apnea Following Bariatric Surgery: 20 Year Outcomes From the Swedish Obese Subjects Study
Source: Obesity (Silver Spring). 2026 Feb 25;34(4):819–28. doi: 10.1002/oby.70154 (PMC13032045; doi:10.1002/oby.70154)

Supplementary appendix

Obstructive Sleep Apnea Following Bariatric Surgery: Twenty-year outcomes from the Swedish Obese Subjects Study

Ida Arnetorp[1], Markku Peltonen[2], Kajsa Sjöholm[1], Per-Arne Svensson[1,3], Peter Jacobson[1], Magdalena Taube[1], Lena M.S. Carlsson[1], Johanna C. Andersson-Assarsson[1*], Sofie Ahlin[1,4*]

[1]Department of Molecular and Clinical Medicine, Institute of Medicine, Sahlgrenska Academy, University of Gothenburg, Gothenburg, Sweden. ida.arnetorp@gmail.com; kajsa.sjoholm@medic.gu.se; per-arne.svensson@medic.gu.se; peter.jacobson@medfak.gu.se; magdalena.taube@wlab.gu.se; lena.carlsson@medic.gu.se; johanna.andersson@medic.gu.se; sofie.ahlin@gu.se;

[2]Finnish Institute for Health and Welfare, Helsinki, Finland. markku.peltonen@thl.fi

[3]Institute of Health and Care Sciences, Sahlgrenska Academy, University of Gothenburg, Gothenburg, Sweden

[4]Region of Västra Götaland, NU hospital group, Department of Clinical Physiology, Trollhättan, Sweden

*Equal contribution

Table of Contents

	Page
**Supplementary Figure S1. Recruitment of study participants to the SOS study.**	2
**Supplementary Table S1. Number of participants at each follow-up time point, stratified by OSA status at baseline, included in the current analysis.**	3
**Supplementary Figure S2. Remission of OSA in men and women over 20 years in the SOS study.**	4
**Supplementary Figure S3. New-onset of OSA in men and women over 20 years in the SOS study.**	5

Supplementary Figure S1. Recruitment of study participants to the SOS study.

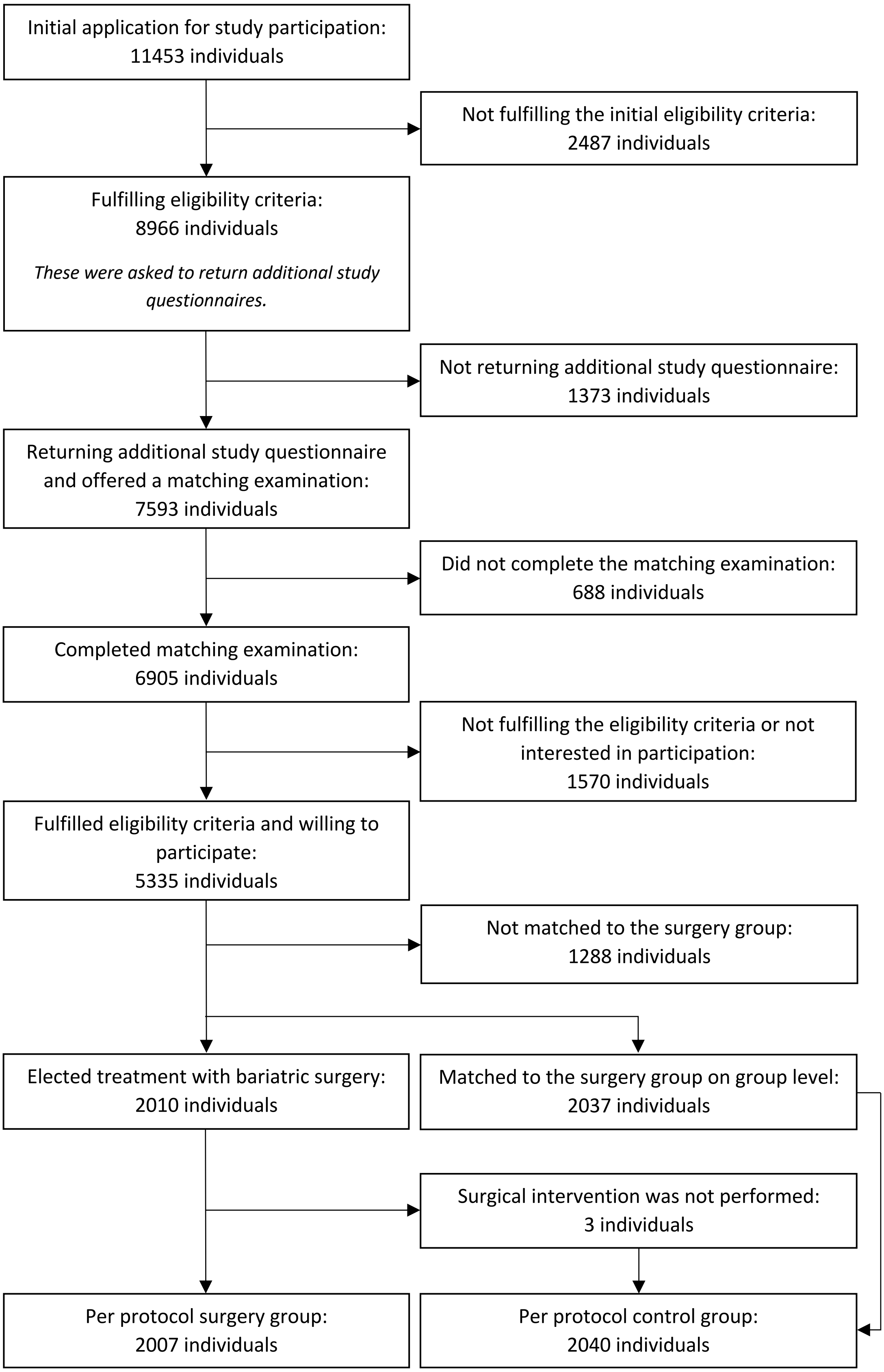

Supplementary Table S1. Number of participants at each follow-up time point, stratified by OSA status at baseline, included in the current analysis.

Time point	Surgery	Control
	Participants with OSA at baseline	
0	500	453
1	439	384
2	421	346
3	379	325
4	370	310
6	343	268
8	320	229
10	316	239
15	214	130
20	143	85
	Participants without OSA at baseline	
0	1505	1582
1	1406	1347
2	1366	1264
3	1229	1164
4	1197	1106
6	1121	1022
8	1055	938
10	1073	924
15	661	530
20	447	332

Supplementary Figure S2. Remission of OSA in men and women over 20 years in the SOS study. Adjusted proportions and 95% CI for OSA at each follow-up timepoint among men and women with OSA at baseline, respectively. Comparison between surgery and control group using a random-effect logistic regression model adjusted for baseline age, BMI, and daily smoking, and inclusion year.

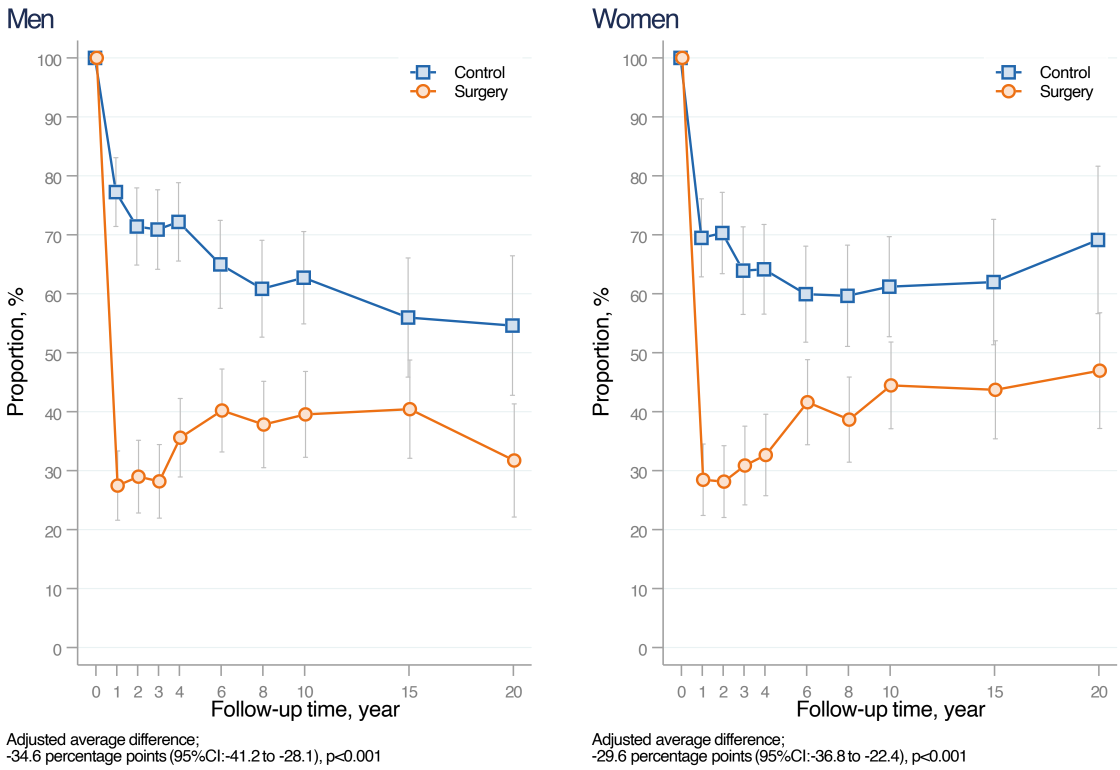

Supplementary Figure S3. New-onset of OSA in men and women over 20 years in the SOS study. Adjusted proportions and 95% CI for OSA at each follow-up timepoint among men and women without OSA at baseline, respectively. Comparison between surgery and control group using a random-effect logistic regression model adjusted for baseline age, BMI, and daily smoking, and inclusion year.

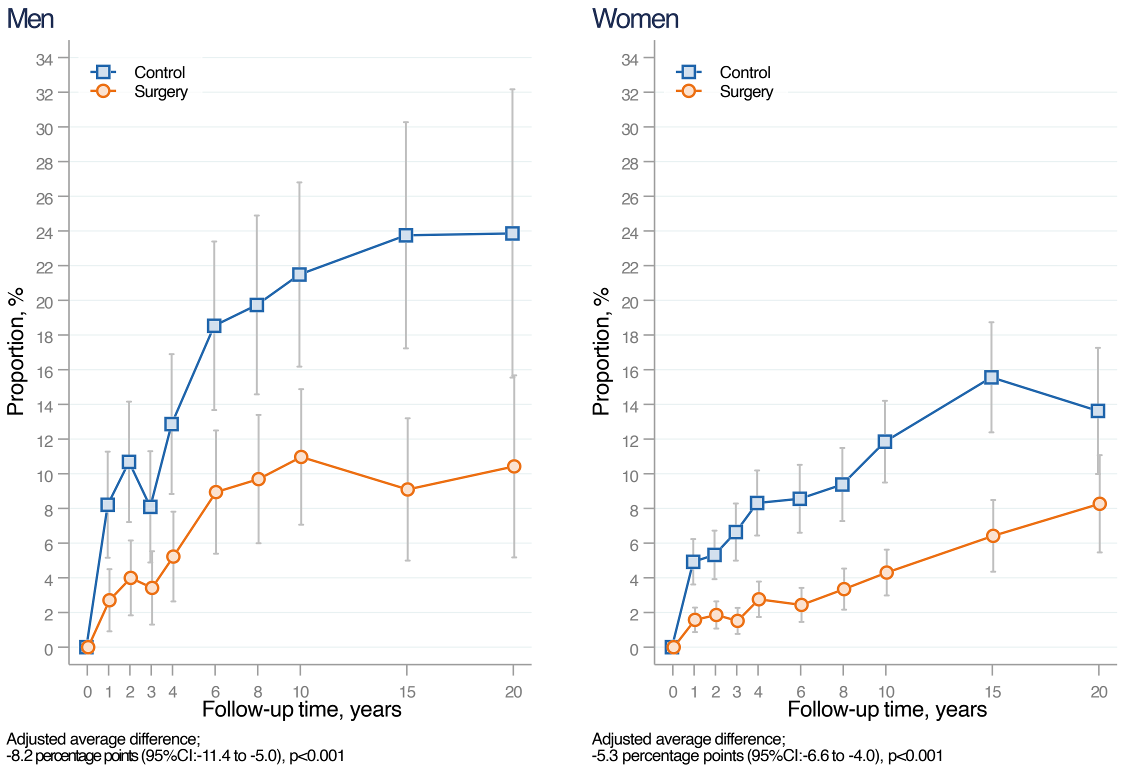

Supplement: Supplementary file 1 — Data S1: Supporting Information. [file OBY-34-819-s001.pdf]
